# WHO RECOMMENDS 29 WAYS TO STOP SURGICAL INFECTIONS AND AVOID SUPERBUGS

**Published:** 2017-01

3 NOVEMBER 2016 | GENEVA - People preparing for surgery should always have a bath or shower but not be shaved, and antibiotics should only be used to prevent infections before and during surgery, not afterwards, according to new guidelines from WHO that aim to save lives, cut costs and arrest the spread of superbugs.

The “Global Guidelines for the Prevention of Surgical Site Infection” includes a list of 29 concrete recommendations distilled by 20 of the world’s leading experts from 26 reviews of the latest evidence. The recommendations were also published today in “The Lancet Infectious Diseases” and are designed to address the increasing burden of health care associated infections on both patients and health care systems globally.

“No one should get sick while seeking or receiving care,” said Dr Marie-Paule Kieny, WHO’s Assistant Director-General for Health Systems and Innovation. “Preventing surgical infections has never been more important but it is complex and requires a range of preventive measures. These guidelines are an invaluable tool for protecting patients.”

Surgical site infections are caused by bacteria that get in through incisions made during surgery. They threaten the lives of millions of patients each year and contribute to the spread of antibiotic resistance. In low- and middle-income countries, 11% of patients who undergo surgery are infected in the process. In Africa, up to 20% of women who have a caesarean section contract a wound infection, compromising their own health and their ability to care for their babies.

But surgical site infections are not just a problem for poor countries. In the United States, they contribute to patients spending more than 400 000 extra days in hospital at a cost of an additional US$ 900 million per year.

**Preventing infections before, during and after surgery**

The guidelines include 13 recommendations for the period before surgery, and 16 for preventing infections during and after surgery. They range from simple precautions such as ensuring that patients bathe or shower before surgery and the best way for surgical teams to clean their hands, to guidance on when to use antibiotics to prevent infections, what disinfectants to use before incision, and which sutures to use.

“Sooner or later many of us will need surgery, but none of us wants to pick up an infection on the operating table,” said Dr Ed Kelley, Director of WHO’s Department of Service Delivery and Safety. “By applying these new guidelines surgical teams can reduce harm, improve quality of life, and do their bit to stop the spread of antibiotic resistance. We also recommend that patients preparing for surgery ask their surgeon whether they are following WHO’s advice.”

No international evidence-based guidelines had previously been available and there are inconsistencies in the interpretation of evidence and recommendations in existing national guidelines. The new WHO guidelines are valid for any country and suitable to local adaptations, and take account of the strength of available scientific evidence, the cost and resource implications, and patient values and preferences. They complement WHO’s popular “Surgical Safety Checklist”, which gives a broad range of safety measures, by giving more detailed recommendations on preventing infections.

**Halting the spread of antibiotic resistance**

Importantly, the guidelines recommend that antibiotics be used to prevent infections before and during surgery only, a crucial measure in stopping the spread of antibiotic resistance. Antibiotics should not be used after surgery, as is often done.

Antibiotics are medicines used to prevent and treat bacterial infections. Antibiotic resistance occurs when bacteria change in response to the use of these medicines. Resistance develops naturally over time, but misuse of antibiotics in humans and animals is rapidly accelerating the process.

Antibiotic resistance is putting the achievements of modern medicine at risk. Without effective antibiotics for the prevention and treatment of infections, organ transplants, cancer chemotherapy and surgeries such as caesarean sections and hip replacements become much more dangerous. This leads to longer hospital stays, higher medical costs, and increased mortality.

Many studies show that implementing a range of preventive measures significantly reduces harm from surgical site infections. A pilot study in 4 African countries showed that implementing a selection of the new recommendations could result in a 39% reduction in surgical site infections. Building upon these successful examples, WHO is developing a guide and toolkit that will help national and local authorities to put the recommendations into action.

Available from: http://www.who.int/mediacentre/news/releases/2016/recommendations-surgical-infections/en/

